# High Occurrence of Shiga Toxin-Producing *Escherichia coli* in Raw Meat-Based Diets for Companion Animals—A Public Health Issue

**DOI:** 10.3390/microorganisms9081556

**Published:** 2021-07-21

**Authors:** Andrea Treier, Roger Stephan, Marc J. A. Stevens, Nicole Cernela, Magdalena Nüesch-Inderbinen

**Affiliations:** Institute for Food Safety and Hygiene, Vetsuisse Faculty, University of Zurich, 8057 Zürich, Switzerland; andrea.treier@uzh.ch (A.T.); stephanr@fsafety.uzh.ch (R.S.); marc.stevens@uzh.ch (M.J.A.S.); n.cernela@access.uzh.ch (N.C.)

**Keywords:** raw meat, pet food, dogs, Shiga toxin, *Escherichia coli*, public health

## Abstract

Feeding pets raw meat-based diets (RMBDs) is becoming increasingly popular but comes with a risk of pathogenic bacteria, including Shiga toxin-producing *Escherichia coli* (STEC). In humans, STEC may cause gastrointestinal illnesses, including diarrhea, hemorrhagic colitis (HC), and the hemolytic uremic syndrome (HUS). The aim of this study was to evaluate commercially available RMBDs with regard to the occurrence of STEC. Of 59 RMBD samples, 59% tested positive by real-time PCR for the presence of Shiga toxin genes *stx1* and/or *stx2*. STECs were recovered from 41% of the 59 samples, and strains were subjected to serotyping and virulence gene profiling, using whole genome sequencing (WGS)-based methods. Of 28 strains, 29% carried *stx2a* or *stx2d*, which are linked to STEC with high pathogenic potential. Twenty different serotypes were identified, including STEC O26:H11, O91:H10, O91:H14, O145:H28, O146:H21, and O146:H28, which are within the most common non-O157 serogroups associated with human STEC-related illnesses worldwide. Considering the low infectious dose and potential severity of disease manifestations, the high occurrence of STEC in RMBDs poses an important health risk for persons handling raw pet food and persons with close contact to pets fed on RMBDs, and is of concern in the field of public health.

## 1. Introduction

Feeding companion animals raw meat has become increasingly popular among cat and dog owners aiming to provide their pets with a natural and healthy diet [1,2]. Raw meat-based diets (RMBDs), also known as Biologically Appropriate Raw Food (BARF), include uncooked raw muscle meats, organ meats, and meaty bones of livestock or wild animals, and are mostly based on the by-products of animals slaughtered for human consumption [3,4]. Since RMBDs are not cooked or pasteurized, concerns have been raised regarding bacterial contamination and the possible transmission of pathogens to pets and humans [5,6,7]. Enterobacteriaceae are the most frequently recovered bacteria from commercially available RMBDs, with a high proportion of sampled RMBDs failing to meet the microbiological standards set out by EC regulation no.1069/2009 in the EU for animal by-products intended for pet food, or the threshold levels for raw human meat products which apply in North America [2,7,8,9]. Of particular concern, Shiga toxin-producing *Escherichia coli* (STEC) were identified in 4% of commercially available RMBDs in the US [10], and contaminated RMBDs have been associated with an outbreak of human STEC infections in the UK [11].

Human infection with STEC is a gastrointestinal illness which may include mild-to-severe non-bloody or bloody diarrhea (BD), hemorrhagic colitis (HC), and the life-threatening hemolytic uremic syndrome (HUS) [12]. STECs are characterized by the proliferation of one or two different types of Shiga toxin encoded by *stx* genes designated *stx1* and *stx2*, with three *stx1* (*stx1a*, *stx1c* and *stx1d*) and ten *stx2* (*stx2a*-*stx2k*) subtypes described so far [13,14]. STECs harboring *stx2a*, *stx2c*, and *stx2d* tend to be associated with severe disease, whereas STECs carrying *stx2b* and *stx2e* are linked to mild clinical symptoms or asymptomatic fecal carriage [15,16,17]. Furthermore, STEC strains may feature additional genes encoding toxins and adherence factors that influence their pathogenic potential, such as *astA* (enteroaggregative *E. coli* heat-stable toxin 1), *cdt* (cytolethal distending toxin), *efa* (enterohemorrhagic *E. coli* factor for adherence), *ehxA* (enterohemolysin), *iha* (IrgA homolog adhesin), *lpf* (long polar fimbriae), *saa* (STEC autoagglutinating adhesin), and *subAB* (subtilase cytotoxin) [18,19,20].

STEC belonging to various serotypes within the O157, O26, O103, O91, and O145 serogroups constitute the main STEC associated with human infections in the EU and in Switzerland and are considered a major concern to human health in Europe [19,21].

Worldwide, STEC causes an estimated 2.8 million acute illnesses and 3890 HUS cases annually, representing a major public health issue [22]. Although frequently linked to foodborne outbreaks, a majority of STEC infections remain sporadic and are significantly associated with consuming undercooked or raw meat, person-to-person transmission, or contact with animals or their environment [23,24]. Although studies demonstrating the occurrence of STEC in RMBDs are rare, they raise the question on the safety of raw pet food and the level of pathogenic potential of STEC occurring in RMBDs [2,10,11].

The aim of this study was to assess the occurrence of STEC isolated from commercially available raw pet food in Switzerland and to characterize the strains by using whole genome analyses.

## 2. Materials and Methods

### 2.1. Sample Collection

During September 2018 and May 2020, a total of 59 RMBD products were purchased from ten different suppliers (designated A–J) either on site in pet-food stores or from certified Swiss RMBD producing enterprises, or in online stores of suppliers located in Switzerland and Germany. The products were purchased frozen or shipped frozen to the laboratory and stored until analysis, according to the recommendations of the suppliers.

The tested products contained either pure muscle or pure organ meat, mixed muscle and organ meat products, or meat supplemented with plant ingredients. Details are listed in Supplementary Table S1.

Products were categorized into those originating from of beef cattle, poultry, horse, lamb, rabbit, venison, and fish. Types of meat within these categories included beef (including rumen and liver) (*n* = 17), chicken (*n* = 7), duck (*n* = 1), quail (*n* = 1), turkey (*n* = 5), ostrich (*n* = 1), horse (*n* = 8), lamb (*n* = 6), rabbit (*n* = 4), reindeer (*n* = 1), moose (*n* = 1), unspecified venison (*n* = 2), salmon (*n* = 4), and perch (*n* = 1).

### 2.2. Screening for Stx Genes

Each sample (10g) was enriched at a 1:10 ratio in Enterobacteriaceae enrichment (EE) broth (Becton, Dickinson, Heidelberg, Germany) for 24 h at 37 °C. One loopful of each of the enrichment cultures was cultured on sheep blood agar (Difco™ Columbia Blood Agar Base EH; Becton Dickinson AG, Allschwil, Switzerland), using the streak-plate method. The resulting colonies were washed off with 2 mL 0.85% NaCl. Samples were then screened by real-time PCR for *stx1* and *stx2*, using the Assurance GDS^®^ for Shiga Toxin Genes (Bio Control Systems, Bellevue, WA, USA).

### 2.3. Recovery of STEC

In the event of a *stx*-positive PCR result, one loopful each of the washed-off suspension was streaked onto on three to five STEC Chromagar plate (CHROMagar, Paris, France) and on three-to-five Brolacin agar plates (Bio-Rad, Hercules, CA, USA) to get single colonies. The plates were incubated overnight at 37 °C.

From each plate, 20–180 individual colonies were picked (mauve colonies on STEC Chromagar plates; yellow colonies on Brolacin Agar plates) and suspended in 0.5 mL 0.85% NaCl. The suspensions were pooled in groups of ten colonies to simplify the screening process. The pooled suspensions were screened for *stx1 and stx2* genes by real-time PCR (LightCycler R 2.0 Instrument, Roche Diagnostics Corporation, Indianapolis, IN, USA), using the QuantiFast Multiplex PCR Kit (Qiagen, Hombrechtikon, Switzerland) according to the guidelines of the European Union Reference Laboratory [25]. In the event of a positive PCR result for *stx1* or *stx2*, the pool was taken apart and the ten colonies were tested individually. From plates yielding more than one *stx* positive colony, one presumptive STEC isolate was randomly chosen for subsequent characterization.

### 2.4. DNA Extraction and Whole Genome Sequencing

The strains were grown on sheep blood agar at 37 °C overnight prior to DNA isolation, using the DNA blood and tissue kit (Qiagen, Hombrechtikon, Switzerland). The DNA libraries were prepared by using a Nextera DNA Flex Sample Preparation Kit (Illumina, San Diego, CA, USA). Whole-genome sequencing was performed on an Illumina MiniSeq Sequencer (Illumina, San Diego, CA, USA). The Illumina-reads files passed the standard quality checks, using the software package FastQC 0.11.7 (Babraham Bioinformatics, Cambridge, UK), and were assembled by using the Spades 3.14.1–based software Shovill 1.0.4 [26,27], using default settings. The assembly was filtered, retaining contigs >500 bp and annotated by using the NCBI prokaryotic genome annotation pipeline [28]. Stx types were determined by an in silico PCR, using the perl script in_silico_pcr (https://github.com/egonozer/in_silico_pcr, accessed on 20 January 2021), using the option “-m, allow one mismatch” and primer sets described in the EU Reference Laboratory for *E. coli* manual for *stx* genes detection [29]. The O and H-types were identified by using SerotypeFinder 2.0 [30]. The virulence gene profiles and antimicrobial resistance genes were determined by using VirulenceFinder 2.0 [31] and Resistance Gene Identifier (RGI) 4.2.2 [32].

The sequence types (STs) of each strain were determined based on seven housekeeping genes, using the tool “MLST” (https://github.com/tseemann/mlst, accessed on 20 January 2021), using PubMLST as the database (https://pubmlst.org/, accessed on 20 January 2021) [33]. The isolates were compared by using core genome MLST (cgMLST) analyses comprising 2513 loci of *E. coli*, using the Ridom SeqSphereC+ software (version 5.1.0; Ridom GmbH, Münster, Germany). Minimum spanning tree (MST) were generated for visualization of strain relatedness, and the threshold for cluster identification was ≤10 alleles between a pair of isolates, according to the Ridom SeqSphereC+ software.

## 3. Results

### 3.1. Real-Time Screening for Stx Genes and Isolation of STEC

**Figure 1:**
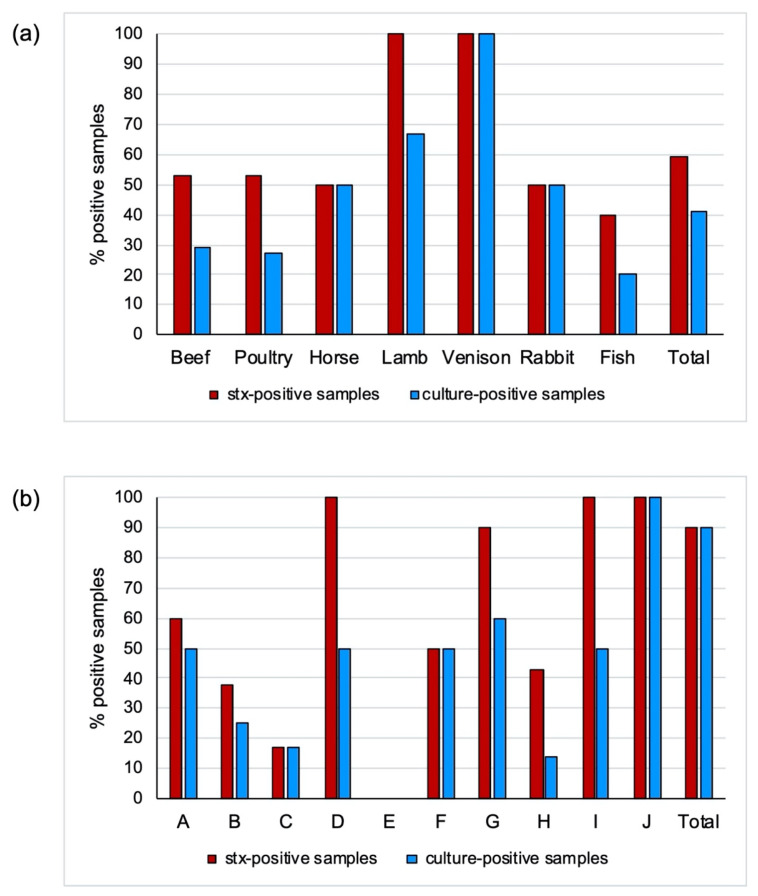
Screening by PCR for Shiga toxin (*stx*) genes and recovery of Shiga toxin-producing *Escherichia coli* (STEC) from 59 samples of RMBDs for pets. (**a**) Percent RMBDs with *stx1* and *stx2* genes detected in enrichment broth cultures obtained from 59 samples, and percent STEC culture-positive RMBDs. (**b**) Percent of RMBDs of ten different suppliers A–J containing *stx* genes and percent of RMBDs contaminated with STEC.

**Figure 2:**
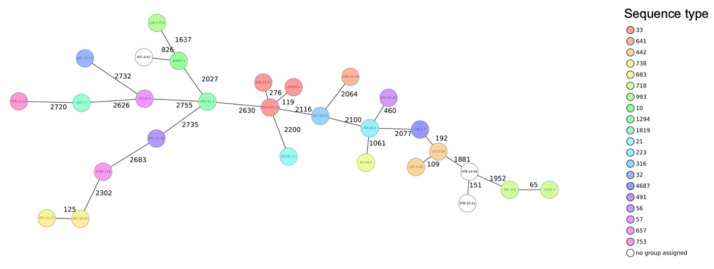
Phylogenetic relationship of 28 Shiga toxin-producing *Escherichia coli* (STEC) isolated from RMBD for pets based on their multilocus sequence type (MLST) allelic profiles. The minimum spanning tree was generated by using SeqSphere (Ridom GmbH). Numbers on connecting lines indicate the number of allele differences between two strains. The colors of the circles represent STs according to the Warwick scheme (http://enterobase.warwick.ac.uk, accessed on 22 January 2021). Strain IDs are indicated in the circles.

By real-time PCR screening of enrichment cultures, *stx1* and/or *stx2* were detected in 35 (59%) of the 59 raw pet-food samples analyzed in this study. Thereof, the majority (*n* = 32) contained *stx2* alone or in combination with *stx1*. The distribution of *stx* genes among the different types and categories of meat is shown in Table 1 and Figure 1. RMBDs containing *stx* genes were detected in products from nine of ten suppliers (Figure 2). STEC was isolated from 24 of the 35 samples with presumptive presence of STEC, corresponding to a recovery rate of 69% and an overall STEC prevalence of 41%. Three samples (beef RMBD samples AT 15 and LS 01, and venison RMBD sample AT 11, respectively) contained two or more distinct STEC strains (Table 2). A total of 28 STEC strains were retrieved. The types and categories of meat from which STEC-positive samples were recovered are shown in Table 1 and Figure 1.

### 3.2. Serotypes and Stx Subtypes

Overall, 20 different serotypes were identified among the 28 STEC (Table 2). Serotypes occurring more than once were O91:H14 (*n* = 3), O146:H21 (*n* = 3), O76:H19 (*n* = 2), O113:H21 (*n* = 2), O146:H28 (*n* = 2), and O168:H8 (*n* = 2). All other serotypes were identified in one strain each (Table 2). No STEC O157:H7 was found. Subtyping of the *stx* genes revealed that six (21%) carried *stx1* genes only: *stx1a* (*n* = 2) and *stx1c* (*n* = 4). Fourteen (50%) carried *stx2* genes only: *stx2a* (*n* = 1), *stx2b* (*n* = 6), *stx2d* (*n* = 5), *stx2e* (*n* = 2), and *stx2g* (*n* = 1). Eight (29%) harbored combinations of *stx1* and *stx2* genes (Table 2).

A total of eight (29% of all the strains) carried subtypes linked to high pathogenic potential, namely *stx2a* (*n* = 3) or *stx2d* (*n* = 5), which were detected among STEC serotypes O168:H8 (*n* = 2), O183:H18 (*n* = 1), O91:H10 (*n* = 1), O113:H21 (*n* = 2), O26:H11 (*n* = 1), and O113:H4 (*n* = 1) originating from beef (*n* = 3), duck (*n* = 1), rabbit (*n* = 1), moose (*n* = 1), salmon (*n* = 1), and turkey (*n* = 1). (Table 2). Fourteen (50%) isolates harbored the low pathogenic subtypes *stx2b* and *stx2e* and were associated with various serogroups, as listed in Table 2. STEC O168:H8 (strain ID LSC 6-3) isolated from a beef sampled harbored both *stx2b* and *stxd* (Table 2).

### 3.3. Additional Virulence Factor Genes

Besides Shiga toxin genes, a number of further toxin genes were identified among the strains, including *astA* (*n* = 8), *cdtB* (*n* = 1), *ehxA* (*n* = 16), *senB* (*n* = 9), and *subA* (*n* = 17) (Table 2). The *eae* gene encoding intimin was detected in two (7%) strains and was associated with STEC O145:H28 harboring *stx1a* (strain ATC 15–17 isolated from beef RMBD), and STEC O26:H11 carrying *stx1a* and *stx2a* (strain ATC 45-11isolated from turkey RMBD) (Table 1). Genes encoding other virulence factors thought to be involved in adhesion to the human intestine included *cif* (*n* = 2), *espI* (*n* = 7), *espP* (*n* = 6), *hra* (*n* = 7), *iha* (*n* = 25), *ompT* (*n* = 21), and *papC* (*n* = 3) (Table 2). None of the strains contained the adherence factors *eidG* and *saa*. Additionally, *epeA* (*n* = 2), *fyuA* (*n* = 3), *gad* (*n* = 25), *ireA* (*n* = 16), *iss* (*n* = 23), *iucC* (*n* = 9), *iutA* (*n* = 9), *katP* (*n* = 3), *kpsE* (*n* = 7), *lpfA* (*n* = 19), *sitA* (*n* = 6), *terC* (*n* = 28), and *traT* (*n* = 25) were found.

### 3.4. Sequence Types and Phylogenetic Relationship

Multilocus sequence typing assigned the strains to 20 different STs. One STEC O123:H16 and two STEC O76:H19 (strains ATB 4-67, ATB 10-31, and ATB 14-66, respectively) were not assigned to any ST (Table 2). STs occurring more than once were ST33 (*n* = 3), ST442 (*n* = 2), ST718 (*n* = 2), and ST738 (*n* = 2). The remaining STs occurred only once (Table 2 and Supplementary Table S2).

**Figure 3:**
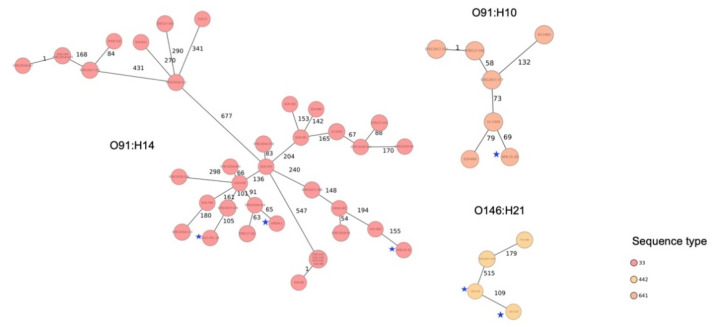
Minimum-spanning trees based on cgMLST allelic profiles of six Shiga toxin-producing *Escherichia coli* (STEC) isolated from RMBD for pets and genome-sequenced clinical STEC available in the database of the Swiss National Reference Centre for Enteropathogenic Bacteria and *Listeria* (NENT) in Switzerland. The numbers on connecting lines represent the number of allelic differences between two strains. Strains from this study are indicated with a star.

The population structure of the strains was visualized by constructing a phylogenetic tree based on cgMLST. The isolates grouped according to serotypes and STs, but they were phylogenetically clearly distinct from each other, with ≥5 different alleles between each pair of neighboring isolates (Figure 2). The genomes of strains belonging to ST33, ST442, and ST641 were compared with the available genomes of corresponding STs present in the database of the Swiss National Reference Centre for Enteropathogenic Bacteria and *Listeria* (NENT) which collects all STEC strains from confirmed human cases nationwide and performs Illumina-based whole-genome sequencing. The cgMLST-based phylogenetic trees are shown in Figure 3 and details are available in Supplementary Table S3). None of the STEC strains belonging to ST33, ST442, or ST642 clustered with a strain in the database, thereby ruling out a direct match with any STEC of those STs reported from a case of human disease in Switzerland.

### 3.5. Transmissible Antimicrobial Resistance Genes

Among the 28 STEC strains, a total of six (21%) carried multiple transmissible AMR (Table 2). Genes encompassed aminoglycoside resistance genes *aac(3)-IIe* (*n* = 1), *aadA* (*n* = 1), *aph(3′)-Ia* (*n* = 1), *aph(3″)-Ib* (*n* = 2), and *aph(6)-Id*) (*n* = 4); sulphonamide resistance genes *sul1* (*n* = 1) and *sul2* (*n* = 4); trimethoprim resistance gene *drfA1*(*n* = 1); and tetracycline resistance *genes tet(A)* (*n* = 1) and *tet(B*) (*n* = 3). Two strains (ATB 4-67 and ATB 6-118, respectively) carried the ß-lactamase gene *bla*_TEM-1_. The distribution of transmissible AMR genes among the STEC strains is given in Table 2.

## 4. Discussion

While recent years have seen a rise in popularity of feeding pets RMBDs, there is rising concern that this trend may come with the risk of exposure to zoonotic pathogens, including STECs. STECs constitute part of the flora of the gastrointestinal tract of a variety of healthy domestic and wild animals and may therefore contaminate meat during slaughter, evisceration, processing, and packing [1,2,34].

In this study, the presence of *stx1* and *stx2* genes was detected in 59% of the enrichment cultures, indicating that the overall contamination of STEC among RMBDs is high. In the majority (69%) of the *stx*-positive samples, the isolation of STEC strains confirmed the presence of the *stx* genes in viable bacterial cells. With an overall prevalence of 41%, the level of STEC contamination in the present study is considerably higher than what another study found previously, where STEC was isolated from 4% of raw pet food in the US [10]. It is also higher than the prevalence of 14% reported during an investigation of raw meat for dogs in the UK [11]. However, comparative data are still scarce, and discrepancies between results of different studies may be due to differences in the testing methodologies. Nevertheless, the present study provides evidence that the occurrence of STEC in RMBDs may currently be underestimated.

The pathogenic potential of STEC may vary according to the presence or absence of a variety of virulence factors. Thus, in addition to serotyping, comprehensive virulence gene profiling is important for risk assessment.

Whole-genome sequencing revealed a high diversity of serotypes that included STEC O26:H11, O91:H10, O91:H14, O145:H28, O146:H21, and O146:H28, which are within the most common non-O157 serogroups associated with human illness in Europe and Switzerland [19,21,35]. The most frequently identified serotypes in the present study were O91:H14 and O146:H21. STEC O91:H14 is a causative agent of mild-to-severe diarrhea and abdominal pain [21,36,37,38], whereas O146:H21 is also found in healthy human carriers [39].

Notably, the serotype most frequently associated with HC and HUS, STEC O157:H7, was not detected in the present study, indicating that STEC O157:H7 may not be considered a suitable marker for STEC detection in RMBDs.

In our study, we found that 29% of the STEC isolated from RMBD harbored *stx2a* or *stx2d*, which are the *stx* subtypes that have the strongest association with HUS [14].

Other toxin genes, including *ehxA* and *subA* found in 57% and 61% of the STEC in this study, are considered important virulence markers for STEC pathogenesis and are frequently detected among human clinical isolates [21,35].

Despite the vast majority (93%) of the strains being negative for the *eae* gene, 25 of the 28 strains harbored *iha*, which is thought to contribute to pathogenicity of *eae*-negative STEC by facilitating attachment to intestinal cells [40]. Taken together, our data indicate that the STECs occurring in RMBDs have the potential to cause disease in humans.

By contrast, STEC-related illness appears to be rare in companion animals [41]. However, there are several studies that provide evidence for the intestinal carriage of STEC in dogs and cats, highlighting their potential epidemiological role as a source for human STEC infections [42,43,44,45]. Hence, it is possible that pets fed RMBDs contaminated with STEC could serve as asymptomatic shedders through their feces, transmitting the pathogen to humans and into the environment.

Interestingly, ten (36%) strains harbored one or more virulence factors, namely *fyuA*, *kpsE*, and *papC*, which are characteristic of extra-intestinal pathogenic *E. coli* (ExPEC), including uropathogenic *E. coli* (UPEC) [46,47,48]. STEC/ExPEC hybrid strains are rarely reported, but, nevertheless, they must be considered high-risk pathogens due to the possibility of a systemic infection in combination with gastrointestinal disease [49]. Furthermore, *papC* and *fyuA* are also prevalent among *E. coli* causing urinary tract infection (UTI) in cats and dogs [50,51]. Therefore, our data indicate that a subset of STEC present in RMBDs may have the potential to cause disease in pets as well as in humans.

Finally, in this study, six (21%) strains, including one STEC/ExPEC strain, carried two or more transmissible AMR genes, predominantly genes conferring resistance to aminoglycosides, which are antimicrobials categorized by the World Health Organization (WHO) as critically important in human medicine [52]. These findings are consistent with previous data that document the presence of AMR genes on mobile genetic elements in STEC [53]. AMR in STEC is worrisome because of the likelihood of horizontal transfer of resistance genes to other pathogens. In view of the ongoing global antimicrobial resistance problem, feeding RMBD to dogs that are undergoing antimicrobial treatment should be reconsidered in order to avoid selection and dissemination of AMR bacteria.

## 5. Conclusions

This study identified commercially available RMBDs as a potential source of STEC, including strains with serotypes, *stx* subtypes, and other virulence traits that are associated with human disease, such as BD, HC, and HUS. In view of the low infectious dose and potential severity of disease manifestations, the high occurrence of potentially harmful STEC in RMBDs represents a risk of infection for persons handling raw pet food and for persons with close contact to pets fed on RMBDs. Our data provide further evidence for the public health risks of raw feeding and highlight the importance of promoting awareness among veterinary and public-health agencies, RMBD suppliers, and pet owners, with the need to focus on safe and hygienic handling of RMBD to protect human and animal health.

## Figures and Tables

**Table 1:** Detection of *stx* genes by PCR and isolation of STEC strains from RMBDs for pets.

		Molecular Detection of *stx* Genes				Isolation of STEC Strains ^a^	
			No. of Samples (%) Positive for				
Type of Meat	No. of Samples	No. of *stx*-Positive Samples (%)	*stx1*	*stx2*	*stx1* and *stx2*	No. of STEC-Positive Samples (%)	No. of STEC Isolated
Beef	17	9 (53)	0 (0)	0 (0)	9 (53)	5 (29)	8
Chicken	7	3 (43)	1 (14)	1 (14)	1 (14)	1 (14)	1
Duck	1	1 (100)	0 (0)	0 (0)	1 (100)	1 (100)	1
Horse	8	4 (50)	0 (0)	2 (25)	2 (25)	4 (50)	4
Lamb	6	6 (100)	1 (17)	0 (0)	5 (83)	4 (67)	4
Moose	1	1 (100)	0 (0)	1 (100)	0 (0)	1 (100)	1
Ostrich	1	1 (100)	0 (0)	0 (0)	1 (100)	1 (100)	1
Perch	1	0 (0)	0 (0)	0 (0)	0 (0)	0 (0)	0
Quail	1	0 (0)	0 (0)	0 (0)	0 (0)	0 (0)	0
Rabbit	4	2 (50)	0 (0)	0 (0)	2 (50)	2 (50)	2
Reindeer	1	1 (100)	0 (0)	1 (100)	0 (0)	1 (100)	1
Salmon	4	2 (50)	1 (25)	0 (0)	1 (25)	1 (25)	1
Turkey	5	3 (60)	0 (0)	0 (0)	3 (60)	1 (20)	1
Venison	2	2 (100)	0 (0)	1 (50)	1 (50)	2 (100)	3
Total	59	35 (59)	3 (5)	6 (10)	26 (44)	24 (41)	28

^a^ PCR positive samples were further cultured and at least one STEC was isolated by growth on Brolacin STEC agar or CHROMagar™. For details, see text.

**Table 2:** Characteristics of 28 STEC isolated from RMBD for pets.

					Shiga Toxin Genes		Intimin		Transmissible Antimicrobial Resistance Genes	
Sample ID	Strain ID	Type of Meat	Serotype	ST	*stx1*	*stx2*	*eae*	Other Virulence Factor Genes		Accession NO.
AT 41	ATC 41-3	Ostrich	O9:H30	1294	*-*	*stx2g*	-	*capU, gad, ompT, papA_F12, papC, terC, traT*	-	JAETYL000000000
AT 45	ATC 45-11	Turkey	O26:H11	21	*stx1a*	*stx2a*	+	*astA, cif, efa1, espA, espB, espF, espJ, espP, fyuA, ehxA, gad, iha, irp2, iss, iucC, iutA, katP, lpfA, nleA, nleB, nleC, ompT, terC, tir, toxB, traT*	*aac(3)-IIe, bla*_TEM-1_	JAETYS000000000
AT 11	ATB 11-12	Venison	O27:H30	753	-	*stx2b*	-	*air, chuA, eilA, gad, iha, ireA, iss, ompT, subA, terC, traT*	-	JAETXZ000000000
AT 11	ATC 11-10	Venison	O54:H45	491	-	*stx2b*	-	*astA, chuA, fyuA, ehxA, gad, iha, ireA, irp2, iss, ompT, papC, pic, senB, sitA, subA, terC, traT, vat, yfcV*	-	JAETYH000000000
AT 10	ATB 10-31	Lamb	O76:H19	nd	*stx1c*	*stx2b*	-	*ehxA, gad, iha, ireA, kpsE, lpfA, pic, senB, sitA, subA, terC, traT*	-	JAETXY000000000
AT 14	ATB 14-66	Lamb	O76:H19	nd	*stx1c*	*-*	-	*ehxA, gad, ireA, kpsE, lpfA, pic, senB, subA, terC, traT*	-	JAETYA000000000
AT 15	ATB 15-29	Beef	O91:H10	641	*-*	*stx2d*	-	*espI, gad, iha, ireA, iss, lpfA, ompT, papC, terC*	-	JAETYB000000000
AT 23	ATB 23-31	Lamb	O91:H14	33	*stx1a*	*stx2b*	-	*espI, ehxA, gad, iha, ireA, iss, iucC, iutA, lpfA, ompT, sitA, subA, terC, traT,*	-	JAETYD000000000
AT 29	ATB 29-1	Lamb	O91:H14	33	*stx1a*	*stx2b*	-	*espI, ehxA, gad, iha, ireA, iss, iucC, iutA, katP, lpfA, ompT, senB, sitA, subA, terC, traT*	-	JAETYF000000000
LS 01	LSB1 P21-24	Beef	O91:H14	33	*stx1a*	*stx2b*	-	*espI, ehxA, gad, iha, ireA, iss, iucC, iutA, katP, lpfA, ompT, senB, sitA, subA, terC, traT*	-	JAETZB000000000
LS 02	LSB 2-27b	Rabbit	O100:H30	993	-	*stx2e*	-	*astA, gad, iss, terC, traT*	-	JAETYV000000000
AT 47	ATB 47-1	Salmon	O113:H4	10	-	*stx2d*	-	*astA, espI, gad, iha, iss, terC*	*aph(3″)-Ib, aph(6)-Id, sul2, tet(B)*	JAETYG000000000
AT 44	ATC 44-40	Moose	O113:H21	56	-	*stx2d*	-	*espP, gad, iha, hra, iss, lpfA, ompT, terC, traT*	-	JAETYM000000000
AT 46	ATC 46-2	Rabbit	O113:H21	223	-	*stx2a*	-	*epeA, espP, ehxA, gad, iha, iss, lpfA, ompT, subA, terC, traT*	-	JAETYT000000000
AT 04	ATB 4-67	Beef	O123:H16	nd	*stx1a*	-	-	*afaA, afaB, afaC, afaD, afaE8, cdtB, espP, gad, hra, iha, iss, iucC, iutA, ompT, terC, traT*	*sul1, aadA, aph(3′)-Ia, aph(3″)-Ib, aph(6)-Id, bla*_TEM-1_, *dfrA1, sul2, tet(A)*	JAETYK000000000
AT 15	ATC 15-17	Beef	O145:H28	32	*stx1a*	-	+	*astA, chuA, cif, espA, espB, espF, espJ, espP, ehxA, gad, iha, iss, neuC, nleA, nleB, nleC, ompT, terC, tir, toxB, traT*	-	JAETYI000000000
LS 01	LSC 1-58	Beef	O146:H21	442	*stx1c*	*stx2b*	-	*espI, ehxA, gad, iha, ireA, iss, iucC, iutA, kpsE, lpfA, ompT, senB, subA, terC, traT*	-	JAETYZ000000000
LS 05	LSC 5-20	Horse	O146:H21	442	stx1c	-	-	*ehxA, gad, iha, ireA, iss, iucC, iutA, kpsE, lpfA, ompT, senB, subA, terC, traT*	-	JAETYY000000000
LS 01	LSC 1-7	Beef	O146:H21	4687	stx1c	-	-	*ehxA, gad, iha, ireA, iss, iucC, iutA, kpsE, lpfA, ompT, senB, subA, terC, traT*	-	JAETZA000000000
AT 21	ATC 21-17	Venison	O146:H28	738	-	*stx2b*	-	*astA, chuA, hra, iha, ireA, iss, lpfA, ompT, subA, terC, traT, usp*	-	JAETYJ000000000
AT 20	ATC 20-47	Horse	O146:H28	738	-	*stx2b*	-	*astA, chuA, hra, iha, ireA, iss, lpfA, ompT, subA, terC, traT, usp*	-	JAETYP000000000
AT 39	ATC 39-3	Horse	O155:H21	683	-	*stx2e*	-	*astA, gad, iha, iss, lpfA, ompT, sepA, terC, traT*	-	JAETYR000000000
AT 49	ATC 49-13	Reindeer	O162:H7	316	-	*stx2b*	-	*ehxA, gad, iha, ireA, iss, lpfA, ompT, subA, terC, traT*	-	JAETYN000000000
LS 06	LSC 6-3	Beef	O168:H8	718	-	*stx2b/2d*	-	*gad, iha, hra, lpfA, terC, traT*	*aph(6)-Id, aph(3″)-Ib, sul2, tet(B)*	JAETZC000000000
AT 09	ATC 9-6	Duck	O168:H8	718	-	*stx2d*	-	*gad, hra, iha, lpfA, terC, traT*	*aph(3″)-Ib, aph(6)-Id, sul2, tet(B)*	JAETYO000000000
AT 07	ATC 7-7	Horse	O166:H28	1819	*stx1c*	*stx2b*	-	*air, chuA, eilA, ehxA, gad, iha, hra, ireA, iss, iucC, iutA, kpsE, ompT, senB, sitA, subA, terC, traT*	-	JAETYU000000000
AT 36	ATC 36-6	Chicken	O176:H4	57	*stx1c*	-	-	*chuA, espI, fyuA, ehxA, gad, iha, ireA, irp2, iss, kpsE, subA, terC*	-	JAETYQ000000000
AT 06	ATB 6-118	Beef	O183:H18	657	*stx1a*	*stx2a*	-	*chuA, cvaC, epeA, espP, ehxA, iha, iss, lpfA, ompT, subA, terC, traT*	*aph(3″)-Ib, aph(6)-Id, bla*_TEM-1_	JAEUYO000000000

Abbreviations: nd, not determined; ST, sequence type; +, presence of gene(s); -, absence of gene(s).

## Data Availability

This whole-genome shotgun project was deposited at DDBJ/ENA/GenBank under the accession numbers JAETXY000000000–JAEUYO000000000. The versions described in this paper are versions JAETXY000000000.1–JAEUYO000000000.1 (https://www.ncbi.nlm.nih.gov/nuccore, accessed on 1 July 2021). Raw sequence data are also available in the Sequence Read Archive (SRA) of the NCBI under BioProject no. PRJNA694525 (https://www.ncbi.nlm.nih.gov/sra/, accessed on 1 July 2021).

## Supplementary Materials

The following are available online at https://www.mdpi.com/article/10.3390/microorganisms9081556/s1. Table S1: Sample analysis. Table S2: Phylogenetic analysis I. Table S3: Phylogenetic analysis II.
